# Multi-platform Affinity Proteomics Identify Proteins Linked to Metastasis and Immune Suppression in Ovarian Cancer Plasma

**DOI:** 10.3389/fonc.2019.01150

**Published:** 2019-11-01

**Authors:** Johannes Graumann, Florian Finkernagel, Silke Reinartz, Thomas Stief, Dörte Brödje, Harald Renz, Julia M. Jansen, Uwe Wagner, Thomas Worzfeld, Elke Pogge von Strandmann, Rolf Müller

**Affiliations:** ^1^Biomolecular Mass Spectrometry, Max-Planck-Institute for Heart and Lung Research, Bad Nauheim, Germany; ^2^German Centre for Cardiovascular Research (DZHK), Partner Site Rhine-Main, Max-Planck-Institute for Heart and Lung Research, Bad Nauheim, Germany; ^3^Center for Tumor Biology and Immunology (ZTI), Philipps University, Marburg, Germany; ^4^Clinic for Gynecology, Gynecological Oncology and Gynecological Endocrinology, Center for Tumor Biology and Immunology (ZTI), Philipps University, Marburg, Germany; ^5^Institute of Laboratory Medicine, Universities of Giessen and Marburg Lung Center (UGMLC), German Center for Lung Research (DZL), Philipps University, Marburg, Germany; ^6^Clinic for Gynecology, Gynecological Oncology and Gynecological Endocrinology, University Hospital of Giessen and Marburg (UKGM), Marburg, Germany; ^7^Institute of Pharmacology, Biochemical-Pharmacological Center (BPC), Philipps University, Marburg, Germany; ^8^Department of Pharmacology, Max-Planck-Institute for Heart and Lung Research, Bad Nauheim, Germany; ^9^Experimental Tumor Biology, Clinic for Hematology, Oncology and Immunology, Center for Tumor Biology and Immunology (ZTI), Philipps University, Marburg, Germany

**Keywords:** ovarian carcinoma, affinity proteomics, proximity extension assay (PEA), SOMAscan aptamer assay, metastasis, SPINT2

## Abstract

A central reason behind the poor clinical outcome of patients with high-grade serous carcinoma (HGSC) of the ovary is the difficulty in reliably detecting early occurrence or recurrence of this malignancy. Biomarkers that provide reliable diagnosis of this disease are therefore urgently needed. Systematic proteomic methods that identify HGSC-associated molecules may provide such biomarkers. We applied the antibody-based proximity extension assay (PEA) platform (Olink) for the identification of proteins that are upregulated in the plasma of OC patients. Using binders targeting 368 different plasma proteins, we compared 20 plasma samples from HGSC patients (OC-plasma) with 20 plasma samples from individuals with non-malignant gynecologic disorders (N-plasma). We identified 176 proteins with significantly higher levels in OC-plasma compared to N-plasma by PEA (*p* < 0.05 by *U-*test; Benjamini-Hochberg corrected), which are mainly implicated in immune regulation and metastasis-associated processes, such as matrix remodeling, adhesion, migration and proliferation. A number of these proteins have not been reported in previous studies, such as BCAM, CDH6, DDR1, N2DL-2 (ULBP2), SPINT2, and WISP-1 (CCN4). Of these SPINT2, a protease inhibitor mainly derived from tumor cells within the HGSC microenvironment, showed the highest significance (*p* < 2 × 10^−7^) similar to the previously described IL-6 and PVRL4 (NECTIN4) proteins. Results were validated by means of the aptamer-based 1.3 k SOMAscan proteomic platform, which revealed a high inter-platform correlation with a median Spearman ρ of 0.62. Likewise, ELISA confirmed the PEA data for 10 out of 12 proteins analyzed, including SPINT2. These findings suggest that in contrast to other entities SPINT2 does not act as a tumor suppressor in HGSC. This is supported by data from the PRECOG and KM-Plotter meta-analysis databases, which point to a tumor-type-specific inverse association of *SPINT2* gene expression with survival. Our data also demonstrate that both the PEA and SOMAscan affinity proteomics platforms bear considerable potential for the unbiased discovery of novel disease-associated biomarkers.

## Introduction

Ovarian cancer (OC) is the deadliest of all gynecological malignancies with >60,000 new cases annually in the United States and the European Union and an overall 12-year survival rate of <20% (1). Six major ovarian cancer types are recognized by the WHO, with high-grade serous carcinoma (HGSC) representing the most common ovarian malignancy. The majority of HGSC patients present with advanced stage disease, tumor masses in the abdomen beyond the pelvis as well as large volumes of cancer-promoting ascites, contributing to a disastrous prognosis (2). A lack of suitable methods for the early detection of HGSC centrally contributes to this dire situation. Furthermore, even though most HGSCs are highly responsive to chemotherapy, the vast majority of patients suffers relapses due to transient or acquired chemoresistance (3), and reliable methods for the detection of recurrent cancer at an early stage are, in exacerbation, missing. The discovery of novel molecules associated with HGSC beyond what is currently known is accordingly of the utmost importance.

CA125 (Mucin 16, MUC16) and human epididymal protein 4 (HE4; also known as WFDC2, WAP Four-Disulfide Core Domain 2) are the best-studied biomarker for OC, but has failed to substantially improve patient survival (4–9). The application of other biomarkers has not improved performance significantly (4). Recent approaches include the development of multi-marker assays, which achieved a marginal advance (10, 11). However, the FDA approved Overa kit measures a combination of apolipoprotein A-1 (APOA1), CA125, follicle stimulating hormone (FSH), HE4 and transferrin (TF) (12), which achieves a higher sensitivity and specificity than any individual marker evaluated to date, suggesting that multi-marker approaches represent a promising approach, Consistently, Han et al. described that the inclusion of E-cadherin (CDH1) and IL-6 improved the performance of CA125 and HE4 (13). Nevertheless, considering the clinical needs further improvements are urgently required, emphasizing the necessity for systematic studies aimed at the identification of novel proteins associated with OC.

Advances in proteomic technologies have improved the identification of biomarker candidates, but systematic or high-throughput analyses have not been described for OC. A particular interesting development in this context is affinity proteomics, including the antibody-based proximity extension assay (PEA) platform (14) offered by Olink and the aptamer-based SOMAscan technology (15) commercialized by SomaLogic. PEA uses pairs of oligonucleotide-coupled antibodies binding epitopes in close proximity on the target protein. As a result, the covalently coupled oligonucleotides anneal to form a template for proximity-dependent DNA polymerization, subsequently amplified by quantitative polymerase chain reaction (qPCR) (14). In contrast, SOMAscan utilizes slow off-rate modified aptamers (SOMAmers), which are short, single-stranded DNA molecules selected for their ability to bind specific proteins with low dissociation rates (15). These features enable their use in quantification assays without the requirement for a second ligand. To achieve greater diversity and high affinity, SOMAmers include non-natural bases harboring functional groups mimicking amino acid side chains.

These affinity proteomics platforms share characteristics that give them a strong advantage over mass spectrometry (MS) in their applicability to unfractionated plasma and related fluids in spite of the massive dynamic range of protein concentrations caused by analytes such as albumin and globulins (16–20). Another advantage is the comparatively simple parallelization, which is required when dealing with heterogeneous human cohorts and large sample numbers. Here, we applied the PEA technology to test its performance for the identification of novel HGSC-associated plasma proteins, and SOMAscan and ELISA for validation of PEA-based data.

## Materials and Methods

### Patients

Peripheral blood was collected from untreated patients with HGSC or benign gynecologic conditions prior to surgery at Marburg University Hospital (Table S1). Peripheral blood was collected in lithium heparin collection tubes (16 I.E. heparin/ml blood) and diluted with an equal volume of PBS prior to centrifugation and cryo-preservation at −80°C. Samples were thawed for ELISA or preparation of shipment to Olink or SomaLogic on dry ice. The collection and the analysis of plasma samples materials were approved by the ethics committee at Philipps University (reference number 205/10). Donors provided written consent in accordance with the Declaration of Helsinki.

### PEA-Based Analysis of Plasma Samples (Olink)

Twenty samples of plasma from patients with HGSC (OC-plasma) and 20 samples of plasma from patients with non-malignant diseases (N-plasma) were randomized in 96-well plates and covered with MicroAmp Clear Adhesive Film (Thermo Fisher Scientific) for PEA analysis at Olink (14, 21). To calculate intra- and inter-assay coefficients of variation (%CV), a pool of randomly selected plasma samples was used. All plasma samples underwent one freeze–thaw cycle prior to proteomic analysis. Three hundred sixty eight markers in four 92-multiplex immunoassay panels (CVD II, Dev, Neuro I, Onc II; Table S2) (details on https://www.olink.com/products/complete-protein-biomarkers-list/) were measured simultaneously for each sample. The Olink assay is based on the proximity extension assay (PEA) technology (14) using pairs of oligonucleotide-labeled antibodies as probes. These paired antibodies bind to the target protein in the sample in close proximity, allowing for the formation of a PCR template by a proximity-dependent DNA polymerization, which is subsequently amplified by quantitative polymerase chain reaction (qPCR) using universal primers. Following the digestion of surplus primers, quantification is performed using a microfluidic chip (96.96 Dynamic Array IFC, Fluidigm Biomark), run on a BioMark platform (BioMark HD System). For details see https://www.olink.com/data-you-can-trust/technology/.

### SOMAscan Analysis of Plasma Samples

The 20 OC-plasma and a subset of 10 N-plasma samples measured by PEA were also analyzed by SomaLogic Inc. (Boulder Colorado, USA). Clinical details are summarized in Table S1. Data for 1,305 SOMAmer probes (SOMAscan assay 1.3K) was obtained per sample (Table S4). Proteins detected in OC-plasma samples with signal intensities not significantly different from negative controls (blanks without sample) were excluded from further analyses. As it has been covered in detail before (15, 22–26), we only briefly describe the SOMAscan methodology. Bead-bound, fluorescence-labeled SOMAmers binned corresponding to the abundance of their target in plasma, are incubated in 96-well plates with three dilution bins of EDTA-plasma using dilution factors inversely correlated to the expected target abundance (0.05, 1, 40%). Subsequent to washing steps, proteins in the bead-captured protein/SOMAmer complexes are biotinylated, followed by photocleaving off the beads and pooling of dilution bins. After reimmobilization of the SOMAmer/protein complexes through the biotinylated proteins on streptavidin beads, followed by additional washing, epitope/protein concentration is determined by proxy from hybridization of the eluted fluorescence-labeled SOMAmers to arrays of complementary oligonucleotides. Resulting raw intensities are processed by hybridization normalization, median signal normalization and signal calibration to control for inter-plate differences based on standard samples included on each plate.

### ELISA and ECLIA

CA125 concentrations were quantified by electrochemiluminescence immunoassay (ECLIA) purchased from Roche (Elecsys® CA 125 II) on a Cobas e602 Modular Analyzer (Roche). Other proteins were quantified by ELISA according to the instructions of the respective manufacturer: BCAM (ELH-BCAM-2; BioCat GmbH, Heidelberg, Germany); EPHA2 (ELH-EPHA2-1; RayBiotech Life, Peachtree Corners, GA, USA); GDF15 (DGD150; R&D Systems, Wiesbaden, Germany); IL-6 (Invitrogen-88-7066-22; Thermo Fisher Scientific, Schwerte, Germany); IL-18BP (DBP180; R&D Systems, Wiesbaden, Germany); OPN/SPP1 (DOST00; R&D Systems, Wiesbaden, Germany); SPON1 (CSB-EL022599HU-96; Cusabio, Houston, TX, USA); VEGFA (BMS277-2; Thermo Fisher Scientific, Schwerte, Germany); WFDC2/HE4 (DHE400; R&D Systems, Wiesbaden, Germany); SPINT2 (EK0773-CAP; Boster, Pleasanton, USA); PVRL4/NECTIN4 (DNEC40; R&D Systems, Wiesbaden, Germany).

### Statistical Analyses

Comparative data were statistically analyzed by the Mann–Whitney *U*-test. Nominal *p* values were adjusted for multiple hypothesis by the Benjamini-Hochberg method. Spearman correlations were analyzed using the scipy.stats.spearmanr functions with Python. Boxplots were constructed by the seaborn.boxplot function. Functional annotations were performed by PANTHER gene ontology (GO) enrichment analysis (27) (http://www.http://geneontology.org). In case of redundancies in the search results only the term with the highest enrichment and significance (lowest FDR) was included in Tables 1, 2.

**Table 1:** Gene ontology term enrichment analysis of biological processes for proteins upregulated in HGSC plasma.

**GO biological process**	**Enrichment**	**FDR**
Cell surface receptor signaling pathway (GO:0007166)	4.1	4.7e-27
Regulation of developmental process (GO:0050793)	3.5	3.8e-19
Immune system process (GO:0002376)^*^	3.2	3.8e-18
Cell communication (GO:0007154)	2.3	8.2e-18
Locomotion (GO:0040011)	4.8	1.4e-17
Cell migration (GO:0016477)^**^	5.6	2.9e-17
Cell adhesion (GO:0007155)^***^	5.6	9.4e-17
Response to cytokine (GO:0034097)	5.1	1.0e-16
Regulation of cell proliferation (GO:0042127)	3.8	5.6e-14
Regulation of cell death (GO:0010941)	3.5	4.1e-12
Chemotaxis (GO:0006935)	5.9	1.7e-10
Extracellular matrix organization (GO:0030198)	7.6	2.9e-10

* *n = 73: ADAM8, AZU1, B4GALT1, CCL15, CD177, CD207, CD27, CD38, CD74, CHI3L1, CLEC1B, CLM6, COL1A1, COLEC12, CSTB, CTSC, CTSD, CTSS, CTSV, CTSZ, CXCL16, DLL1, EFNA4, EPHA2, EPHB6, ESAM, FADD, FAS, FAS, FASLG, FSTL3, Gal-1, GP6, GPC1, HAVCR2, ICOSLG, IFNGR1, IGF1R, IL18BP, IL1RT1, IL5RA, IL2-RA, IL6, ITGAV, JAMA, LAIR1, LTBR, SMAD5, MMP9, MUC16, N2DL2, NEP, PDGFRA, PGLYRP1, PRTN3, RET, RETN, S100A11, SCF, ST2, SYND1, TGFR2, TNFR2, TNFRSF21, TNFRSF4, TNFSF13, TNFSF13B, TR, TXLNA, VEGFA, VEGFR2, VIM, VSIG4*.

** *n = 43: ADAM8, AZU1, B4GALT1, CCL15, CD177, CD74, CLEC14A, COL1A1, CXCL16, DDR1, EGFR, EPHA2, EPHB4, ERBB4, ESAM, GDF8, GDNF, GP6, GPC1, HGF, IL6, ITGAV, JAMA, MATN2, MDGA1, MK, MMP9, NOV, NRP2, NTRK2, PDGFRA, PLXNB1, PLXNB3, PODXL, PRTN3, PTPRF, RET, SCF, SKR3, SYND1, TNFRSF12A, VEGFA, VEGFR2*.

*** *n = 41: ADAM8, B4GALT1, BCAM, CADM3, CD177, CDH3, CDH6, CNTN1, CNTN5, DAG1, DDR1, DSC2, EGFR, EPHA2, EPHB4, ESAM, FLRT2, ITGAV, JAMA, MSLN, MUC-16, NID2, NOV, NRP2, PDGFRA, PLXNB3, PODXL, PTPRF, PVR, PVRL4, RET, S100A11, SCARF1, SCARF2, SCF, SIGLEC1, SPON1, TGFR-2, TNFRSF12A, vWF, WISP1*.

*Enrichment: fold increase over random distribution; FDR, false discovery rate. The table lists the top 12 specific, non-redundant terms yielding a minimum enrichment of 2-fold*.

**Table 2:** Gene ontology term enrichment analysis of molecular functions for proteins upregulated in HGSC plasma.

**GO biological process**	**Enrichment**	**FDR**
Protein tyrosine kinase activity (GO:0004713)	13.5	8.6e-10
Glycosaminoglycan binding (GO:0005539)	8.8	4.0e-08
Insulin-like growth factor binding (GO:0005520)	34.9	1.3e-07
Cytokine binding (GO:0019955)	11.4	4.7e-07
Heparin binding (GO:0008201)	9.7	5.6e-07
Extracellular matrix binding (GO:0050840)	18.9	8.3e-07
Cell adhesion molecule binding (GO:0050839)	5.1	1.2e-06
Serine-type endopeptidase activity (GO:0004252)	8.7	5.7e-06
Peptidase inhibitor activity (GO:0030414)	7.2	8.5e-05
VEGF-activated receptor activity (GO:0005021)	69.8	1.5e-04
Integrin binding (GO:0005178)	8.5	2.2e-04
TNF-activated receptor activity (GO:0005031)	54.2	2.8e-04
Semaphorin receptor activity (GO:0017154)	44.3	4.7e-04
Death receptor activity (GO:0005035)	44.3	4.8e-04
Scavenger receptor activity (GO:0005044)	14.1	6.4e-04
Laminin binding (GO:0043236)	21.1	6.3e-04
Collagen binding (GO:0005518)	10.9	2.1e-03
Ephrin receptor activity (GO:0005003)	25.7	2.4e-03
Fibronectin binding (GO:0001968)	17.4	8.2e-03
PDGF receptor binding (GO:0005161)	24.4	2.4e-02

*Enrichment: fold increase over random distribution; FDR, false discovery rate. The table lists the top 20 specific, non-redundant terms yielding a minimum enrichment of 5-fold*.

## Results

### Identification of Proteins Increased in HGSC Plasma

**Figure 1:**
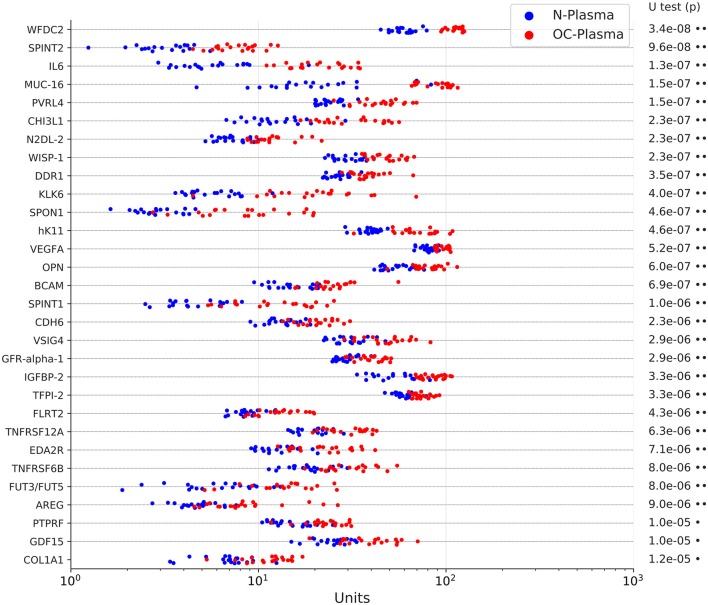
Levels of the top 30 upregulated proteins in OC-plasma (red) vs. N-plasma (blue) based on PEA signals. The dot plots show the results for 20 OC-plasma and 20 N-plasma samples. The indicated *p*-values were determined by Mann-Whitney U test and adjusted for multiple hypothesis testing by Benjamini-Hochberg correction. X-axis units represent normalized intensity PEA signals. Dots next to the *p*-values indicate the extent of significance: ^•^*p* < 1e-3, ^••^*p* < 1e-5.

We first sought to identify proteins present at elevated levels in plasma samples from patients with HGSC vs. non-malignant gynecological diseases by PEA. We selected four disease-centered 92-multiplex panels offered by Olink (CVD II, Dev, Neuro I, Onc II; Table S2) to determine relative protein levels in plasma from 20 untreated FIGO stage III HGSC patients (OC-plasma; Table S1) and from 20 patients with uterine myomatosis, ovarian cysts or endometriosis (N-plasma; Table S1). Of these, 157 proteins were significantly more abundant in OC-plasma as compared to N-plasma (Benjamini-Hochberg-adjusted *p* < 0.05 by U test; ratio OC/N > 1 in Table S3). The data for the 30 top proteins (highest significance) are shown in Figure 1. The only protein completely separating OC-plasma and N-plasma samples was WFDC2, consistent with previous findings (8, 9). Other proteins yielding highly significant differences (adjusted *p* ≤ 1.5 × 10^−7^) between the sample sets were SPINT2 (Serine Peptidase Inhibitor Kunitz Type 2), IL-6 (interleukin 6), MUC16, and PVRL4 (Poliovirus Receptor-Related Protein 4; also known as NECTIN4; Nectin Cell Adhesion Molecule 4). In addition, a number of proteins previously not described in previous studies were also significantly upregulated in OC-plasma, including BCAM, CDH6, DDR1, N2DL-2 (ULBP2), and WISP-1 (CCN4) (Figure 1).

We also found 19 proteins present at significantly higher levels (adjusted *p* < 0.05) in N-plasma relative to OC-plasma (ratio OC/N <1 in Table S3). We did not follow up on these proteins, as the goal of the present study was the identification of markers upregulated in HGSC patients.

### Functions of Upregulated Proteins

Functional annotation of the proteins upregulated in HGSC plasma by gene ontology (GO) term enrichment analysis identified several biological processes known to be critical for HGSC growth and progression (2), including immune regulation, cell adhesion, cell migration, cell proliferation, cell death and extracellular matrix organization (Table 1). The “immune system process” group comprised 73 proteins, the metastasis-related groups “cell migration” and “cell adhesion” 43 and 41 proteins, respectively (listed below Table 1). The most significant molecular functions associated with upregulated plasma proteins were membrane-receptor-driven pathways triggered by interactions with extracellular matrix (ECM) components and growth factors, such as IGF (insulin-like growth factor), VEGF (vascular endothelial growth factor), TNF (tumor necrosis factor), semaphorins, and PDGF (platelet-derived growth factor), as well as extracellular proteases and their inhibitors. These findings are consistent with our knowledge of progression-driving mechanisms in HGSC.

### Correlation of Olink, SOMAscan and ELISA Data

**Figure 2:**
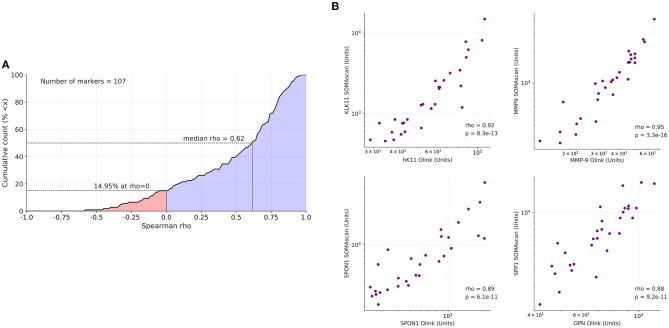
Correlation of PEA with SOMAscan data. **(A)** Data for 107 markers were analyzed to calculate the cumulative distribution of Spearman correlation coefficients (ρ) between PEA and SOMAscan signal intensities, yielding a median value of ρ = 0.62. The analysis was carried out with 20 OC-plasma and 10 N-plasma samples. The light blue area indicates positive correlations (85.05% of all instances), light red indicates negative correlations (14.95%). **(B)** Dot plots showing highly significant positive correlations of PEA and SOMAscan data (*n* = 30) for KLK11, MMP9, SPON1, and SPP1/OPN.

To assess the validity of the results obtained by the antibody-based Olink platform we reanalyzed all samples by the aptamer-based SOMAscan proteomic assay using the 1.3 k panel with 1,305 probes (Table S4). Out of the 157 proteins identified by PEA as upregulated in OC-plasma (see above) 107 were present (by gene names) in the SOMAscan panel. Spearman analysis across all plasma samples revealed a positive median correlation of ρ = 0.62 for these 107 proteins between the platforms (Figure 2A; Table S5), exemplified in Figure 2B for KLK11 (kallikrein 11), MMP9 (matrix metallopeptidase 9, SPON1 (Spondin 1) and OPN (osteopontin), referred to as SPP1 (secreted phosphoprotein 1) in the SOMAscan dataset.

**Figure 3:**
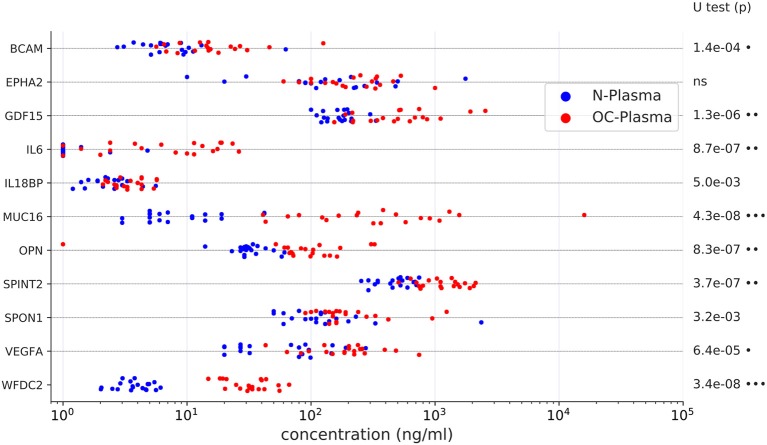
Levels of upregulated proteins in OC-plasma (red) vs. N-plasma (blue) determined by ELISA. Details as in Figure 1. X-axis units represent concentrations (ng/ml) determined by comparison with a calibration curve for the respective protein. Dots next to the *p*-values indicate the extent of significance: ^•^*p* < 1e-3, ^••^*p* < 1e-5, ^•••^*p* < 1e-7.

**Figure 4:**
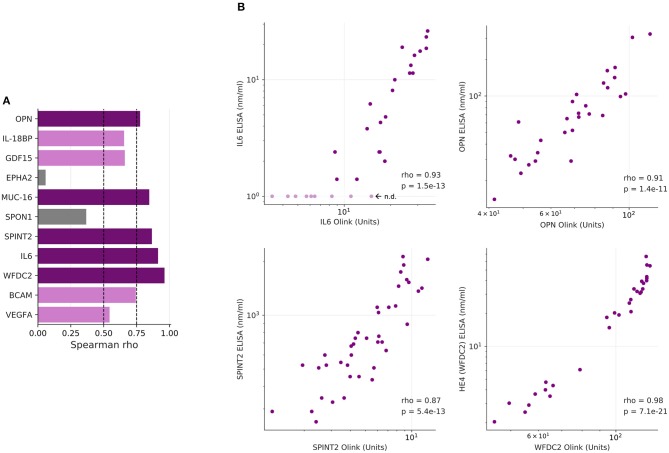
Correlation of PEA and ELISA data. **(A)** Spearman correlations for the markers determined in Figure 3. Dark purple: ρ > 0.75; light purple: 0.75 ≥ ρ > 0.5; gray: 0.5 ≥ ρ > 0. **(B)** Dot plots showing highly significant positive correlations of PEA and SOMAscan data (*n* = 30) for IL-6, OPN, CA125/MUC16, and HE4/WFDC2.

As several relevant markers are not part of the SOMAscan panel (such as MUC16 and WFDC2), we also determined plasma levels for 12 proteins of the Olink dataset by ELISA. The data in Figure 3 show that significant differences between the two plasma sample sets observed by PEA could be reproduced by ELISA for all proteins except EPHA2 (ephrin receptor A2). Consistently, 10 of these 12 proteins correlated between PEA and ELISA data (Spearman ρ > 0.5; Figures 4A,B). It is noteworthy that we observed an excellent correlation for SPINT2 in particular (ρ = 0.87; Figures 4A,B).

### Origin of Upregulated Plasma Proteins

**Figure 5:**
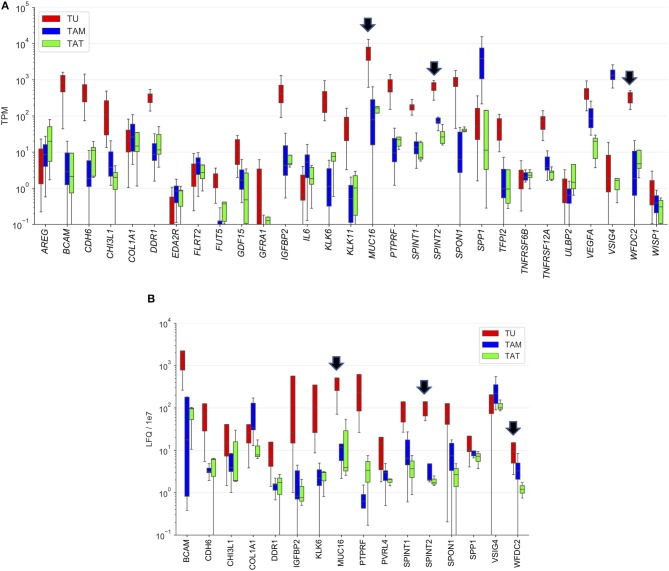
Cellular origin of upregulated proteins. **(A)** Transcriptome analysis of the top 30 proteins increased in OC-plasma (from Figure 1). **(B)** Proteome analysis as in **(A)**. Due to the lower sensitivity of MS-based proteomics, especially for secreted proteins (29), data were not available for a number of proteins. Boxplots show medians (horizontal line in boxes), upper and lower quartiles (box) and range (whiskers). Arrows point out MUC16, SPINT2, and WFDC2. TU, tumor cells; TAM, tumor-associated macrophage; TAT, tumor-associated T-cells.

To identify the cell types producing the proteins upregulated in OC-plasma we made use of our previously generated transcriptome and proteome data for tumor cells, tumor-associated macrophages (TAMs) and tumor-associated T cells (TATs) from HGSC ascites (28, 29). As illustrated in Figure 5A, expression of several genes was highly tumor-cell-specific, including *WFDC2* and *SPINT2*, and slightly less pronounced for *MUC16*. The proteome data generally showed a similar trend as the RNA expression data (Figure 5B). However, a considerably stronger tumor-cell-specificity for SPINT2 was observed compared to WFDC2 and MUC16.

### Association of SPINT2 With a Poor Clinical Outcome of HGSC

**Figure 6:**
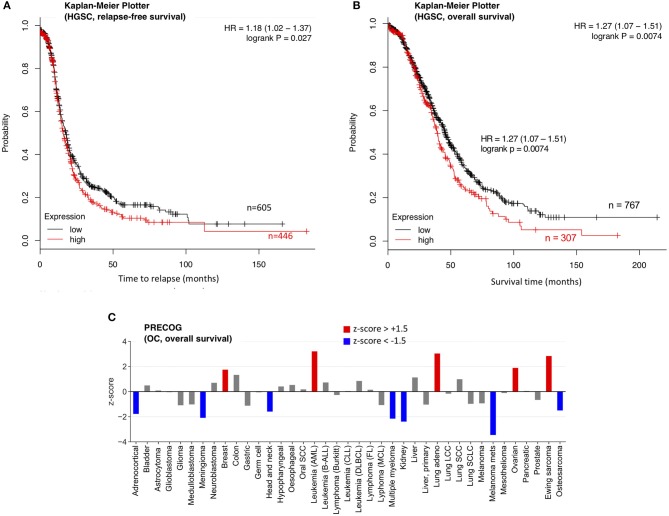
Association of *SPINT2* mRNA expression with survival of OC patients. **(A)** Kaplan-Meier plot for 1074 HGSC patients in the Kaplan–Meier Plotter database (31) (updated version at http://kmplot.com) analyzing the association of *SPINT2* with RFS. HR, hazard ratio. **(B)** Kaplan-Meier plot for 1074 HGSC patients in the same database analyzing the association of *SPINT2* with OS. **(C)** z-scores (PRECOG data) for the association of *SPINT2* with the overall survival (OS) of the indicated tumor entities (32). Red: association with a short OS (z-score above +1.5;). Blue: association with a short OS (z-score below −1.5).

The increased levels of SPINT2 in HGSC plasma and its high expression in tumor cells suggest a tumor-promoting function, which contrasts results published for other entities (30). We therefore analyzed the association of SPINT2 with the clinical outcome of OC. As it was not possible to address this question for SPINT2 plasma levels due to the small size of our cohort, we analyzed two public databases (PRECOG and Kaplan-Meier Plotter) (31, 32) for association of *SPINT2* mRNA expression with relapse-free survival (RFS) and overall survival (OS). The Kaplan-Meier Plotter in Figures 6A,B show that *SPINT2* levels in HGSC tumor tissue are significantly associated with both a short RFS (logrank *p* = 0.027) and a short OS (*p* = 0.0074). This was confirmed by PRECOG data, which also indicate a short OS for OC patients (Figure 6C; z-score = 1.89). Intriguingly, the association with OS appears to be entity-specific. While a strong association with a short survival was also observed for AML, lung adenocarcinoma and Ewing sarcoma, the opposite was true for instance for kidney carcinoma, meningioma, metastatic melanoma and OC (Figure 6C), suggesting that SPINT2 may not be classified as a general tumor suppressor or promoter.

## Discussion

### Identification of Plasma Proteins Upregulated in HGSC Patients and Cross-Validation of Data Obtained by Different Proteomic Methods

Using the antibody-based Olink PEA platform, we identified 176 protein signals significantly different in HGSC-plasma. Using the competing commercial SOMAscan proteomic assay as well as ELISA, the PEA results were independently validated in the same sample sets. Beyond strengthening our initial findings, the parallel employment of two affinity proteomics platforms (PEA and SOMAscan) also lends itself to a limited comparison between them, which, given a strong median correlation (Figure 2A), indicates wide-ranging cross-validation of the platforms, which notably use different molecular classes of affinity reagents (aptamers for SOMAscan and classical antibodies for PEA). We therefore conclude that both affinity proteomics platforms bear great potential for large-scale biomarker discovery studies.

Affinity proteomics (including ELISA)-delivered signals do not necessarily reflect target protein abundance, as potential interference with target/binder interaction through antigen occlusion by single nucleotide polymorphisms, differential splicing, post-translational modifications, complex formation etc. may impact signal strength. Where affinity assays that are likely not targeting identical epitopes cross-validate, the otherwise “epitopomic” nature of the signal may none the less be guardedly read as representing protein abundance difference, as for instance for the 10 out of 12 candidate proteins tested using PEA and ELISA in Figure 4.

### Functions of Plasma Proteins Upregulated in HGSC Patients

According to our GO term analysis the proteins elevated in OC-plasma are mainly associated with receptor tyrosine kinase and ECM-induced signaling impinging on immune cell functions and metastasis-associated processes, such as cell adhesion, motility/migration, cell proliferation and survival as well as ECM reorganization (Table 1). The molecular functions driving these processes are mainly growth factor and cytokine signaling (e.g., IGF, VEGF, TNF, and PDGF pathways), integrin signaling triggered by ECM components (such as collagen, laminin, and fibronectin) and protease activity (Table 2). These mechanisms are known to drive HGSC growth and metastasis (28, 29, 33–35), and therefore suggest that the plasma composition mirrors the pro-tumorigenic HGSC microvenvironment.

This notion is supported by our observation that many proteins driving these biological processes and pathways are actually found at elevated levels in HGSC plasma. Growth factor/cytokines and their receptors with the highest significance (Figure 1) include AREG (amphiregulin), GDF15 (growth differentiation factor 15), GFR-apha-1 (GFRA1; Glial cell line-derived neurotrophic factor receptor), IL-6, OPN (SSP1; osteopontin), TNFRSF6B (decoy receptor for death ligands), and VEGFA. The group of molecules impacting the ECM is comprised of (i) cell adhesion molecules such as BCAM, COLA1A, FLRT2 (fibronectin leucine rich transmembrane protein 2), PVRL4 (NECTIN4) and SPON1 (spondin 1), (ii) proteases, including kallikreins 6, 8, and 11 and (iii) protease inhibitors, e.g., the serine inhibitors SPINT1, SPINT2, TFPI2 (tissue factor pathway Inhibitor 2), and WFDC2 (HE4). Furthermore, a large group of proteins elevated in OC-plasma has functions in immune suppression (*n* = 73; see Table 1), and comprises mostly cytokines, but also other proteins with immune functions, such as VSIG4 (V-set and immunoglobulin domain containing 4), a phagocytic receptor as well as strong negative regulator of T-cell proliferation and IL2 production. Intriguingly, a number of the proteins with increased abundance in OC-plasma are intracellular proteins, which may represent cargo of extracellular vesicles. An example in this context is WISP-1 (CCN4), which is a downstream regulator in the Wnt/Frizzled-signaling pathway.

Several of these proteins have been described in the literature as mediators of ovarian cancer progression, including associations of their expression in tumor tissue or blood or ascites levels with ovarian cancer survival, for instance IL-6, GDF15, OPN/SST1, PVRL4, and VEGFA (13, 28, 36–43). One of the proteins with the most significant difference in Figure 1, i.e., SPINT2, however, has not been linked to HGSC prior to the present study and is discussed in more detail in the subsequent section. Likewise, a number of other proteins found at elevated levels in OC-plasma have not been described previously as upregulated plasma proteins in HGSC, such as N2DL-2 (ULBP2; a ligand of the NKG2D receptor on natural killer cells), WISP-1 (CCN4; a member of the connective tissue growth factor family), DDR1 (a receptor tyrosine kinase interacting with the extracellular matrix), BCAM (basal cell adhesion molecule), and CDH6 (cadherin 6), attesting to the potential of affinity proteomics platforms such as PEA and SOMAscan for biomarker identification from primary clinical material.

A SOMAscan-based analysis of ovarian HGSC serum samples was published very recently, but this work was carried out with a different goal than our study, namely the identification of prognostic biomarkers (44). The authors identified BDNF (brain derived neurotrophic factor) and PDGF (Platelet Derived Growth Factor) molecules as strong predictors of progression-free survival. PDGFA is also present in the PEA panel used in our study and was found to be upregulated in OC-plasma, complementing the observation of Mysona and colleagues (44).

### Role of SPINT2

SPINT2 (also referred to as serine peptidase inhibitor Kunitz type 2) is a transmembrane protein that inhibits a variety of serine proteases (30). One of the proteases targeted by SPINT2 is hepatocyte growth factor (HGF) activator, resulting in a decreased formation of active pro-tumorigenic HGF. SPINT2 also inhibits several other proteases that are relevant in the context of tumorigenesis, such as plasma and tissue kallikrein. The *SPINT2* gene has been proposed to act as a putative tumor suppressor in several cancer entities, including gastric cancer (45), glioblastoma (46), medulloblastoma (47), melanoma (48), and renal cell carcinoma (49). A tumor suppressive role in these tumor types is supported by two types of observation. First, the *SPINT2* promoter is frequently methylated, resulting in downregulation of the *SPINT2* gene by epigenetic silencing (45–48); and second, SPINT2 inhibited the motility and invasion of cancer cells as well as their viability and anchorage independent growth (46–49), in part by inhibiting the activation of HGF as alluded to above. In contrast, invasion by oral squamous carcinoma cells was promoted by SPINT2, and in oral squamous carcinoma tumor tissue HAI-2 immunoreactivity accompanied neoplastic progression with intense staining of invasive tumor cells (50). These findings suggest that SPINT2 has tumor-type-specific functions that may either promote, or, as in the majority of cases analyzed, suppress tumor progression.

Consistent with this conclusion is our analysis of the association of *SPINT2* expression with survival for 39 different cancer types. The data in Figure 6C clearly suggest that a high *SPINT2* expression can either be beneficial or detrimental. OC appears to fall into the category of tumor entities where SPINT2 may have a tumor-promoting rather than suppressive role, which is suggested by two observations. First, SPINT2 is upregulated in the plasma of HGSC patients, as shown by PEA and ELISA in the present study; and second, *SPINT2* expression is associated with a poor clinical outcome in terms of both OS and RFS (Figure 6A). Importantly, this association was observed with datasets from two independent large meta-analyses (Figure 6). Currently, it remains unknown how SPINT2 may promote tumor progression. Addressing this question in future studies could be of great importance to elucidate the mechanisms of HGSC growth and metastasis, and may lead to the discovery of novel functions of SPINT2.

### Potential as Biomarkers

As discussed in the Introduction, a number of the markers we have identified have been proposed previously as OC biomarkers as discussed in the Introduction, including APOA1, CGB, FSHB, IL-6, MMP7, and TF (4, 10–13). We therefore analyzed the performance of these proteins, which are all present in the SOMAscan 1.3k panel, in our patient cohort. The performance of these markers was considerably worse compared to the combinations defined in the present study, since the concentration of each of these markers determined by SOMAscan showed a large overlap of OC-plasma and N-plasma samples (Figure S1). This is surprising since three of these markers, APOA1, FSH, and TF, are part of the FDA-approved Overa (TM) multi-marker panel (12), which achieved a sensitivity of 91.3% (100% with stage III patients) and a specificity of 69.1%. Theses discrepancy may either be due to differences in the patient cohorts, or, more likely, attributable to the different technologies measuring specific epitopes and protein subtypes.

The low prevalence of OC requires a test with a minimum specificity of 99.6% to achieve a low but useful positive predictive value of 10% (51, 52). It also requires sufficient sensitivity to detect tumors smaller than 0.5 cm in diameter if it is to achieve a reduction in mortality of 50% (53). Our own data suggest that such sensitivity may be difficult to achieve. In a screening scenario, the tumor burden is much lower (probably >100-fold) compared to the stage III HGSC patients in the present study (53). The markers measured in our study (including MUC16 and WFDC2) showed, however, a median difference between concentrations in OC- and N-plasma samples clearly lower than 100-fold (Figure 1). On the other hand, the linearity of the relationship between tumor burden and the plasma signals of the markers identified in the present study remains to be explored. This issue therefore remains an open question to be carefully addressed by subsequent studies.

The differential diagnosis of suspected OC or patients monitored for relapse is another application of biomarkers where the requirements for minimum specificity and sensitivity are lower due to higher prior probabilities and increased tumor load. In such a scenario, a successful application of the markers identified in the present study may be realistic. To evaluate the true clinical potential of these markers, evaluation in larger and independent cohorts must be the next step with the goal to develop novel improved multi-marker panels.

## Data Availability Statement

The data supporting the conclusions of this manuscript will be made available by the authors, without undue reservation, to any qualified researcher.

## Ethics Statement

The studies involving human participants were reviewed and approved by Ethics Committee at Philipps University (reference number 205/10). The patients/participants provided their written informed consent to participate in this study.

## Author Contributions

JG, SR, TW, EP, and RM designed the study and coordinated the project. JG, FF, and RM performed the statistical and bioinformatic analyses. JG and RM wrote the manuscript. SR, JJ, and UW recruited patients, collected and processed plasma samples, and revised the manuscript. SR, TS, DB, and HR measured protein concentrations by ELISA and ECLIA.

### Conflict of Interest

The authors declare that the research was conducted in the absence of any commercial or financial relationships that could be construed as a potential conflict of interest.

## Supplementary Material

Figure S1 Performance of proposed HGSC plasma biomarkers. The plots show SOMAscan signals in N-plasma (*n* = 10; blue) and OC-plasma (OC = 20; red) for apolipoprotein A1 (APOA1), choriogonadotropin-β (CGB), follicle-stimulating hormone-β (FSHB), interleukin 6 (IL6), metalloproteinase 7 (MMP7) and transferrin (TF) determined by SOMAscan.

Table S1 Patient characteristics.

Table S2 PEA data for all samples and proteins analyzed.

Table S3 PEA data for proteins up- or downregulated in OC-plasma relative to N-plasma (adjusted *p* < 0.05 by *U*-test).

Table S4 SOMAscan data for all samples and proteins analyzed.

Table S5 Correlation of PEA and SOMAscan data (Spearman ρ).
