# Supplementary material for: Multi-platform Affinity Proteomics Identify Proteins Linked to Metastasis and Immune Suppression in Ovarian Cancer Plasma
Source: Front Oncol. 2019 Nov 1;9:1150. doi: 10.3389/fonc.2019.01150 (PMC6839336; doi:10.3389/fonc.2019.01150)
Supplement: Figure S1 — Performance of proposed HGSC plasma biomarkers. The plots show SOMAscan signals in N-plasma (n = 10; blue) and OC-plasma (OC = 20; red) for apolipoprotein A1 (APOA1), choriogonadotropin-β (CGB), follicle-stimulating hormone-β (FSHB), interleukin 6 (IL6), metalloproteinase 7 (MMP7) and transferrin (TF) determined by SOMAscan. [file Data_Sheet_1.PDF]

Multi-platform affinity proteomics identify proteins linked to metastasis and immune suppression in ovarian cancer plasma

J. Graumann et al.

Supplemental Figure S1

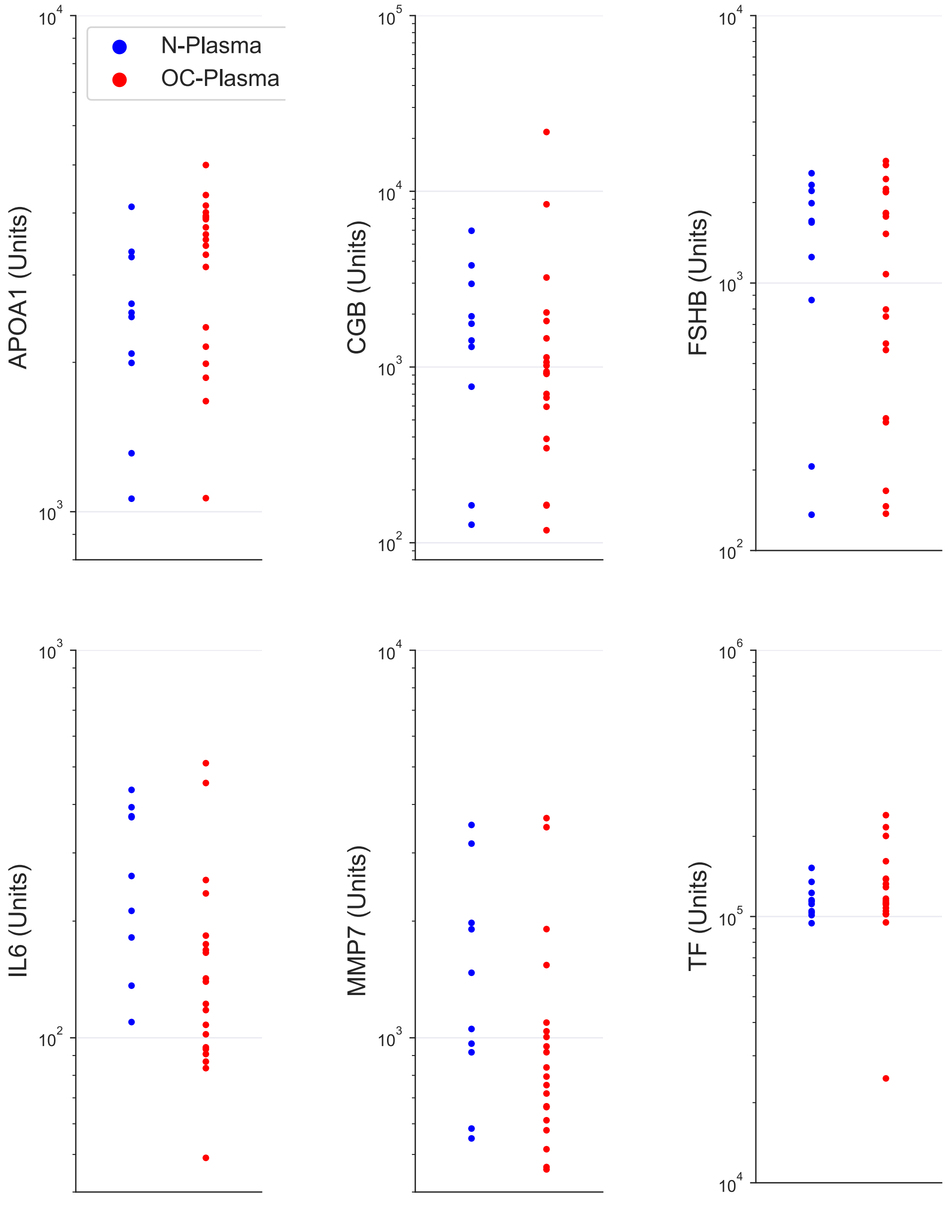

Performance of previously proposed HGSC plasma biomarkers. The plots show SOMAscan signals in N-plasma (n=10; blue) and OC-plasma (OC=20; red) for apolipoprotein A1 (APOA1), choriogonadotropin-β (CGB), follicle-stimulating hormone-β (FSHB), interleukin 6 (IL6), metalloproteinase 7 (MMP7) and transferrin (TF) determined by SOMAscan.
